# High-throughput sequencing reveals the diversity and community structure of rhizosphere fungi of *Ferula Sinkiangensis* at different soil depths

**DOI:** 10.1038/s41598-019-43110-z

**Published:** 2019-04-25

**Authors:** Tao Zhang, Zhongke Wang, Xinhua Lv, Yang Li, Li Zhuang

**Affiliations:** College of Life Sciences, Key Laboratory of Xinjiang Phytomedicine Resource Utilization, Ministry of Education, Shihezi University, Xinjiang Shihezi, 832003 China

**Keywords:** Microbial ecology, Soil microbiology, Microbial ecology

## Abstract

*Ferula sinkiangesis* is a valuable medicinal plant that has become endangered. Improving the soil habitat of *Ferula sinkiangesis* can alleviate plant damage. Fungi play an important role in the soil, but current information on the fungal community structure in the habitat of *Ferula sinkiangesis* and the relationship between soil fungi and abiotic factors remains unclear. In this study, we analyzed the relative abundance of fungal species in the rhizosphere of *Ferula sinkiangesis*. Spearman correlation analysis showed that the abiotic factor total potassium (TK) significantly explained the alpha diversity of the fungal community. At altitude, available phosphorus (AP), nitrate nitrogen (NN) and TK were significantly associated with the fungal species. In addition, a two-way ANOVA showed that soil depth had no significant effects on the alpha diversity of rhizosphere and non-rhizosphere fungi. Interestingly, linear discriminant effect size (LEfSe) analysis indicated that different biomarkers were present at varying soil depths. These findings may be related to the growth and medicinal properties of *Ferula Sinkiangensis*.

## Introduction

More than 150 species of ferula exist globally, mainly in Southern Europe, North Africa Central Asia and their adjacent ares^1^. Approximately 26 species and a single variant are present in China^2^. *Ferula Sinkiangensis* is an early spring ephemeral plant of the Umbelliferae family. It begins to pump stems at the end of March and blooms the end of April. Full bloom occurs in mid-May^3^. At this time, the roots and stems of *Ferula Sinkiangensis* contain large amounts of milky white resin. The resin is termed ferulic and has high medicinal value including anticancer^4,5^, anti-influenza^6,7^ antibacterial^8^, and antioxidant activity^9^. *Ferula sinkiangensis* is the major species of the genus ferula^2^ and was included in the Pharmacopoeia of the People’s Republic of China in 1977^10^. In recent years, *Ferula sinkiangensis* has approached extinction due to natural and human factors^5^. Considering its important medicinal value and endangered status, the protection of *Ferula sinkiangensis* is extremely important.

As the foundation of plant life, soil provides the nutrients for plant growth. Hiltner first coined the term “rhizosphere area” in 1904 to describe the soil affected by rhizosphere sediment exudates, mucus, and exfoliated cells^11,12^. These soils contain energy that can be supplied to microorganisms^12–14^. Diverse microbial communities concentrate in these soils. At the same time, microbes enriched in the roots of the plants contribute to the nutrient absorption and plant growth^15,16^, increasing the stress resistance^16^, and ecological adaptability of the plants. Both benefit from this interaction and participate in the complex rhizosphere ecosystem. In the rhizosphere niche, it is believed that the carbon sources released by plant roots enters the soil food chain through bacterial channels^17,18^. However, studies tracking the fate of root carbon using stable isotope detection (SIP) have shown that rhizosphere microorganisms add recently fixed plant 13 C to fungi to much higher levels than bacteria. This applies not only to mycorrhizal fungi but also to saprophytic fungi^19,20^. In addition, bacteria bind to mycorrhizal fungi and the mycelia of saprophytic fungi, passively dependent on the compounds secreted by the mycelia) and actively (obtaining the energy present in the mycelia) to obtain energy from the fungi^19,21^. It is noteworthy that because the cell walls of fungi vary in composition^22,23^, different bacteria may be selected from different bacterial communities. We may thus have underestimated the role of fungi in the rhizosphere niche of plants, which may explain the lack of studies on *Ferula sinkiangensis* rhizosphere fungi. We believe that this is not conducive to performing research on the rhizosphere ecological mechanisms of *Ferula sinkiangensis* to reveal the cause of extinction. Thus, this study explores the role of fungi in the rhizosphere community structure and provides diversity information on *Ferula sinkiangensis*.

Understanding the relationship between the structural diversity of the *Ferula sinkiangensis* fungal community and other factors is also considered. Biological factors, (plant species, developmental stages, physiological conditions) and abiotic factors (soil chemistry and planting systems) affect the structure and diversity of rhizosphere microbial communities^24–28^. Previous studies have shown that changes in water, nutrient availability, and salinity, regulate plant photosynthetic rates and growth, through altering the composition of rhizosphere bacterial communities through changes in the exudation patterns^29,30^. Marques and coworkers showed that both the growth and genotype of sweet potato affects the structure of the microbial community in the rhizosphere^31^. Ndour and colleagues indicated that the genetic characteristics of pearl millet determined the diversity of rhizosphere bacteria^32^ Studies in natural ecosystems have found that increasing nitrogen (N) reduces the microbial biomass^33–36^. Compost and biochar alter mycorrhization and tomato root exudation^37^. pH also affects the growth of soil microbial communities^38,39^. Root traits and microbial community interactions can be regulated by phosphorus availability and acquisition^40^. Rhizospheric microorganisms may be indirectly or directly affected by these factors, but in most cases can simultaneously exist. Therefore, in a complex habitat, understanding the key factors that drive and maintain microbial diversity can predict the response strategies of the ecosystem to future environmental changes. In this study, our key objective was to understand the fungal community structure and diversity by artificially changing factors that are convenient for human operation. On this basis, we aimed to understand the relationship between fungal communities, diversity, and other factors, so as to indirectly protect and increase plant productivity.

The rapid development of high-throughput sequencing technology has facilitated the diversity and structural analysis of rhizosphere bacterial communities^41,42^. Recording the diversity and richness of the rhizosphere bacterial community, permits an assessment of its contribution to the rhizosphere core, functional bacteria, and flora that changes with geological isolation and soil depth^43^. We used Illumina HiSeq sequencing and multivariate analysis to assess the diversity and structure of the rhizosphere bacterial community in *Ferula sinkiangensis*. To our knowledge, this is the first study that analyzes the diversity and structure of the rhizosphere fungi community in *Ferula sinkiangensis* through high-throughput sequencing.

## Materials and Methods

### Site description and experimental design

During the *Ferula sinkiangensis* growing season (April to May), we collected samples of *Ferula sinkiangensis* (Good growth, no pests and diseases) rhizosphere soil and non-rhizosphere soil from three different locations in Yining city, Xinjiang, China (Table S1). Rhizosphere and non-rhizosphere soil samples were collected using the Riley and Barber’s shaking method^44,45^. In the three different geographical conditions, nine ferula were randomly selected and the entire roots were excavated from the soil profile (Amongst the nine *Ferula sinkiangesis*, every three were divided into a group as a repeating unit). Soil samples were collected at a root depth of 0–5 cm, 5–15 cm and 15–40 cm. Samples were marked using a mixed marker system. An initial set of letters were used to indicate the site number, rhizosphere, and non-rhizosphere (E, R and S represent the rhizosphere areas at sites 1, 2 and 3, respectively), (NE, NR and NS represent the non-rhizosphere areas of sites 1, 2 and 3, respectively). The second number indicated the depth (1, 2 and 3 represent the 0–5, 5–15, and 15–40 cm depths, respectively) and the third number indicates the number of repetitions. For example, E2.3 represents the third replicate soil sample at a depth of 5–15 cm in the *Ferula sinkiangesis* root of the first site.

### DNA extraction, amplification, and sequencing of the ITS rRNA gene

We extracted DNA from each sample using the Centrifugal Soil Genomic DNA Extraction Kit. DNA concentration and purity were monitored on a 1% agarose gel. The DNA was diluted to 1 ng/μL using sterile water. We used the Internal Transcribed Spacer (ITS) 36, technique to analyze nucleic acid sequences. ITS1 is located between 18 S and 5.8 S of the eukaryotic ribosomal rDNA sequence. Because it does not need to be added to mature ribosomes, ITS1 can withstand additional mutations during evolution. Its evolution rate is 10 times that of 18SrDNA, which is moderately conserved. The area can be used to study the classification order below. The ITS rRNA gene was amplified in the ITS1–5F region using ITS5–1737 Forward (GGAAGTAAAAGTCGTAACAAGG),andITS2-2043Reverse(GCTGCGTTCTTCATCGATGC) primers. All PCR reactions were performed using Phusion® High-Fidelity PCR Master Mix (New England Biolabs). The X1 (containing SYB green) buffer was mixed with an equal volume of PCR product and electrophoresed on a 2% agarose gels. Samples with bright master bands between 400–450 bp were selected for further experiments. PCR products were mixed in equal density ratios. PCR products were purified using Qiagen Gel Extraction Kits (Qiagen, Germany). Sequencing libraries were generated using TruSeq DNA PCR-Free Sample Preparation Kits (Illumina, USA) following the manufacturer’s recommendations. Index codes were added. The library quality was assessed on Qubit 2.0 Fluorometer (ThermoScientific) and an Agilent Bioanalyzer 2100 system. Finally, the library was a sequenced on an Illumina HiSeq^46,47^. 2500 platform to generate 250 bp paired-end reads.

### Soil physicochemical properties

The content of organics was determined by external heating with potassium bichromate. Total nitrogen content was determined using the perchlorate-sulfuric acid digestion method, and fox 1035 automatic nitrogen determination apparatus. The content of total phosphorus was determined by acid soluble molybdenum anti-colorimetry and agilent CARY60 UV spectrophotometer. The total kalium content was determined using the acid dissolution - atomic absorption method using the Thermo Scientific Series Atomic Absorption Spectrometer. Nitrate nitrogen and ammonium nitrogen content was determined through the 0.01 M calcium chloride extraction method using a BRAN + LUEBBE flow analyzer. Available phosphorus was determined through extraction with sodium bicarbonate and molybdenum inverse colorimetry. pH measurements were performed using a mettler tolido FiveEasy Plus pH meter.

### Data analysis

Cutadapt^8^ software was used to filter and control data. Low -quality areas were removed and reads that conformed to the length of the target fragment were retained. According to the barcode, sample data were extracted from the reads obtained. Barcode and primer sequences were cut off and the Cut adapt parameter (−q) was set for quality control to obtain raw reads. The original sequencing data possessed a proportion of interference data to allow the results of information analysis to be accurate and reliable. We performed splicing and filtering of the raw datasets to obtain valid data. OUT clustering and species classification analysis were then performed based on valid data.

Alpha Diversity was used to analyze the complexity of species diversity for a sample through six indices^48^, including observed-species, Chao1, Shannon, Simpson, ACE, and Good-coverage. All indices in the samples were calculated with QIIME (Version 1.7.0) and displayed with R software (Version 2.15.3). Chao1 and Abundance Coverage based Estimators (ACE) were selected to identify community richness. Shannon and Simpson indexes were used to identify community diversity. Good coverage was used to characterize the sequencing depth. Observed species were used to directly reflect the rationality of data sequencing, and indirectly reflected the species richness in the sample.

Beta diversity analysis was used to evaluate differences in sample species complexity, Beta diversity on both weighted and unweighted unifrac were calculated using QIIME software (Version 1.7.0). Cluster analysis was preceded by principal component analysis (PCA) which was applied to reduce the dimensions of the original variables using the FactoMineR package and ggplot2 package in R software (Version 2.15.3). Principal Coordinate Analysis (PCoA) was performed to obtain principal coordinates and to visualize complex and multidimensional data. A distance matrix of weighted or unweighted unifrac amongst the samples was obtained prior to being transformed to a new set of orthogonal axes, by which the maximum variation factor was demonstrated by the first principal coordinates, and the second maximum was obtained by the second principal coordinates. PCoA analysis was displayed through the WGCNA package, Stat packages and ggplot2 package in R software (Version 2.15.3). Unweighted Pair-group Methods with Arithmetic Mean (UPGMA) clustering was performed using QIIME software (version 1.7.0) as a hierarchical clustering method to interpret the distance matrix using average linkages. Linear discriminant analysis (LDA) effect sizes (LEfSe) was used for significant differences between statistical groups^49^. At the same time, CCA/RDA/dbRDA analysis and correlation analysis of the diversity index and environmental factors could be combined with environmental factors, and environmental impact factors that significantly affected community changes between the groups.

## Results

### Rhizospheric and non-rhizospheric soil areas share or have specific fungal operational taxonomic units, and the fungal communities are not evenly distributed

**Figure 1:**
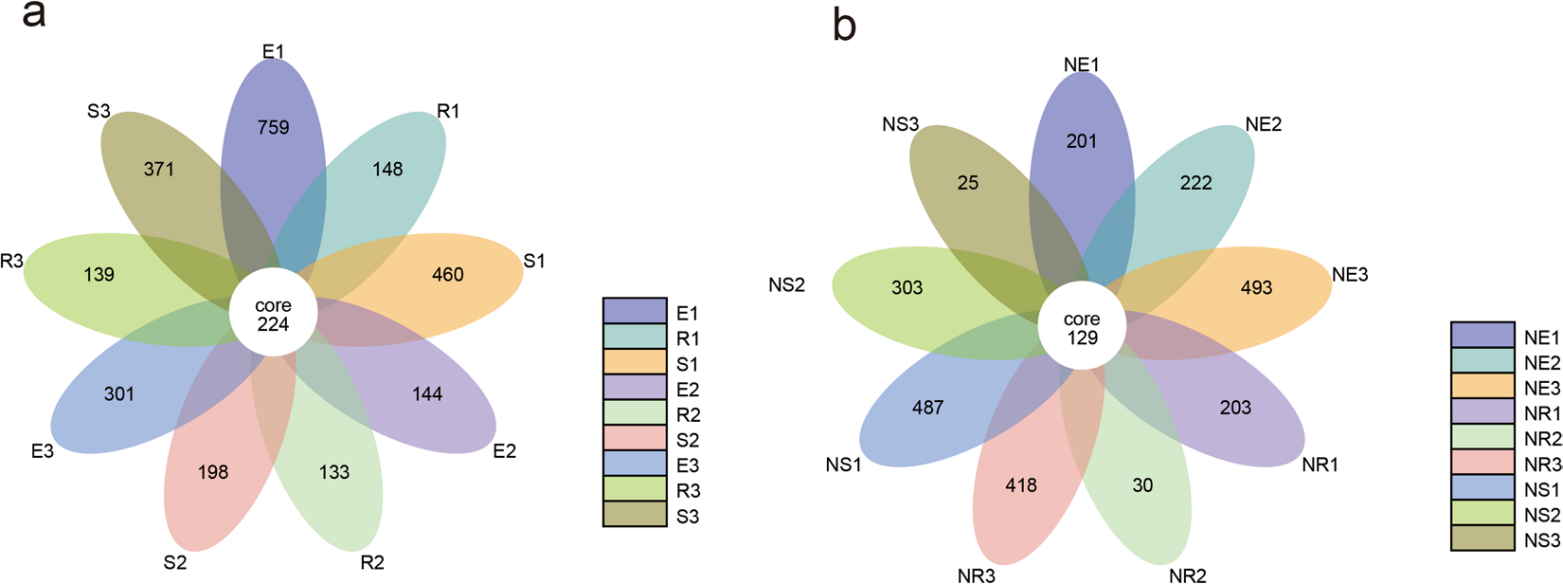
Operational Taxonomic Units (OTU) based petal maps. *Ferula sinkiangesis* rhizospheric (**a**) and non-rhizospheric (**b**) petal maps. Description: Each petal in the diagram represents a (Group) sample. Different colors represent different samples (Groups). Middle core numbers represent the number of OTUs common to all samples, and the number on the petal represents the number of OTUs unique to this sample (Group). E, R, and S represent the rhizosphere of *Ferula sinkiangesis* 1, 2, and 3 plots, respectively. NE, NR, and NS represent the non-rhizosphere of *Ferula sinkiangesis* 1, 2, and 3 plots, respectively. Numbers indicate the root depth (1, 2 and 3 indicate depths of 0–5 cm, 5–15 cm and 15–35 cm, respectively).

Petal maps revealed 224 core OTUs in the soil samples from different locations and depths in the rhizosphere soil region (Fig. 1a), whilst 129 core OTUs samples from different locations and depths were obtained in the non-rhizosphere soil (Fig. 1b). Interestingly, whether in the rhizosphere or non-rhizosphere, the number of unique OTUs for each soil sample differed. Specifically, the number of OTUs in each sample of the rhizosphere region were significantly higher than those of the non-rhizosphere region, and the number of OTUs in many of the soil samples in the non-rhizosphere soil region were extremely low. Rarefaction curves were constructed and founded on the levels of data extracted using the Observed species index and the corresponding number of species (Supplementary Fig. S1). The curve in the Rarefaction diagram tends to be flat, and more data is produced for new OTUs, indicating that the levels of sequencing are reasonable. Good coverage ranged from 99.4 to 99.8% (Supplementary Table S1) and the depth of measurements aligned with all requirements. The Rank Abundance Curve (Supplementary Fig. S2) showed a rapid decline, indicating that only a few dominant fungi account for a large proportion of the total fungal population.

### Soil TK can Significantly explain the alpha diversity of fungal communities

**Figure 2:**
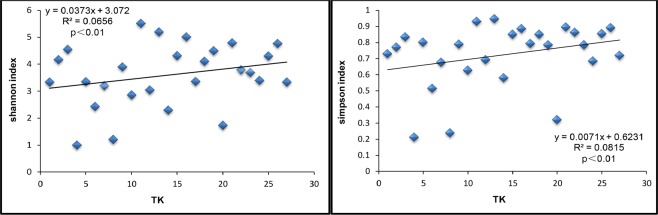
Stepwise multiple linear regression model. Description: The best model for the diversity of the rhizosphere fungi community of *Ferula sinkiangesis* was explained. Multiple nonlinear regressions were performed using the stepwise method. The abiotic factors were used as independent variables, and the Shannon or Chao 1 index were used as dependent variables.

Spearman correlation analysis showed that the abiotic factor TK had a significant and strongly positive correlation with the Shannon and Simpson indexes (Shannon index, R2 = 0.59, simpson index, R2 = 0.57, p < 0.01, Table 1). Conversely, other abiotic Factors (pH, TOC, TN, TP, NN, AN, AP, TS and Altitude) did not exhibit significant correlation with Shannon and Simpson indexes (Table 1). These diversity indices reflect the diversity and uniformity of species communities; the greater the index, the higher the species diversity, the more uniform the distribution. Therefore, TK is accountable for the diversity of fungal communities. simultaneously, the stepwise multiple linear regression model shows that TK is the only important influencing factor among all the abiotic factors listed in the article, affecting the alpha diversity of the rhizosphere community (Shannon index: y = 0.3245 × −1.8459, p < 0.01; simpson index: y = 0.0071 × +0.6231, p < 0.01, Fig. 2).

**Table 1:** Pearson correlation analysis tested the correlation between alpha diversity index and abiotic factors in all samples.

	shannon index	simpson index	chao1 index
pH	−0.131	−0.168	−0.216
TOC	0.114	0.091	0.092
TN	0.144	0.120	−0.002
TP	0.093	−0.023	0.209
TK	0.586**	0.569**	0.365
NN	0.175	0.161	−0.134
AN	0.109	0.286	−0.163
AP	0.335	0.247	0.347
TS	0.275	0.308	0.109
Altitude	−0.012	−0.064	0.320

TS, total salt content;AP, quick-acting phosphorus; AN, ammonium nitrogen; NN, nitrate nitrogen; TK, total kalium content; TP, total phosphorus content; TN, total nitrogen content; TOC, total organic carbon. *p < 0.05, **p < 0.01.

### Dominant fungal phyla and their relationship with abiotic factors

**Figure 3:**
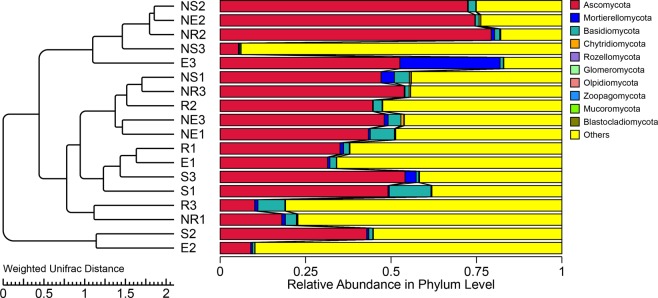
UPGMA distance sample cluster tree. Description: The UPGMA cluster analysis was performed with the Weighted Unifrac distance matrix, and the clustering results were integrated with the species relative abundance of each sample at the Phylum level. The left side is the UPGMA clustering tree structure, and the right side is the species relative abundance distribution of each sample at the phylum level.

Analysis of dominant bacteria in all soil samples found that Ascomycota (42.87%), Basidiomycota (3.21%), Mortierellomycota (2.66%), Chytridiomycota (0.21%), Glomeromycota (0.05%), Rozellomycota (0.04%), Olpidiomycota (0.01%), Zoopagomycota (0.01%), Mucoromycota (0.01%) and Blastocladiomycota (0.01%) are fungi with higher phyla levels. Amongst the ten phyla, Ascomycota, Mortierellomycota and Basidiomycota were relatively richer than other phyla, with Ascomycota having the highest abundance (Fig. 3). Step by step, we found that there is a significant correlation between dominant fungal phyla and abiotic factors (Table 2). As showed in Table 2, pH, TK, NN, AP and TS were markedly correlation with fungal phyla. For example, pH markedly positively correlated with the relative abundances of Basidiomycota and Rozellomycota. TK showed markedly positively relationships with Basidiomycota and Mucoromycota. NN and AP showed obviously positively relationships with Basidiomycota. And TS have important relationships with Mucoromycota (Table 2). Contrary, pH markedly negative correlated with the relative abundances of Mortierellomycota and Olpidiomycota (Table 2). Meanwhile, Kickxellomycota, Zoopagomycota,Glomeromycota, Chytridiomycota and Ascomycota have no significant correlation with various factors. No obviously relationships was found between the relative abundance of fungi and TOC, TN, TP, AN and altitude.

**Table 2:** Pearson correlation analyses testing the relationship between the relative abundance of dominant rhizosphere bacterial phyla and soil properties and non-soil parameters and across sampling sites.

	Ascomycota	Mortierellomycota	Basidiomycota	Chytridiomycota	Glomeromycota	Olpidiomycota	Zoopagomycota	Rozellomycota	Mucoromycota	Kickxellomycota
pH	−0.178	−0.456*	0.393*	−0.048	−0.118	−0.395*	−0.074	0.514**	−0.254	−0.271
TOC	0.180	−0.031	0.294	0.100	0.063	−0.091	0.084	−0.002	0.222	0.020
TN	0.126	0.015	0.368	0.147	0.114	0.071	0.054	0.031	0.158	0.226
TP	0.225	0.215	0.032	0.221	−0.192	−0.058	0.139	−0.061	−0.035	0.077
TK	0.313	0.063	0.471*	0.353	0.355	0.266	0.369	0.136	0.383*	0.218
NN	−0.117	−0.338	0.420*	0.143	−0.361	−0.239	−0.142	0.331	−0.060	−0.241
AN	−0.225	0.022	−0.042	−0.013	0.066	0.228	−0.057	−0.045	0.036	0.296
AP	0.277	0.067	0.390*	0.335	0.214	0.271	−0.014	0.135	0.284	0.206
TS	0.320	0.037	−0.033	−0.057	0.220	0.261	0.256	−0.093	0.593**	0.253
Altitude	0.303	0.361	−0.213	0.003	0.117	0.326	0.021	−0.305	0.133	0.194

^*^p < 0.0 ^**^p < 0.01.

### Dominant fungal genus and their relationship with abiotic factors

**Figure 4:**
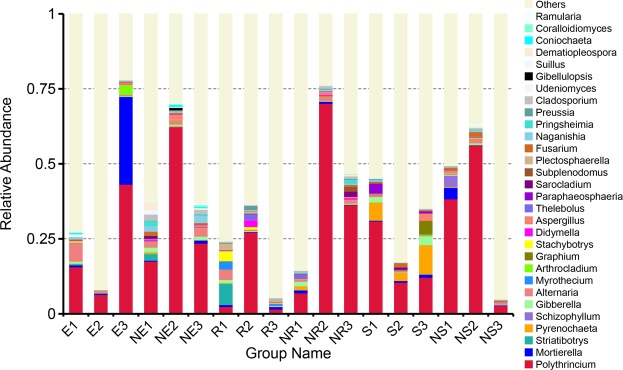
Histogram of relative abundance of rhizosphere microorganisms in *Ferula sinkiangesis*. Description: Relative abundance of the top thirty genera in the rhizosphere and non-rhizosphere of *Ferula sinkiangesis*. Sample names and relative abundance are shown. Others indicate the sum of the relative abundance of all genera except the thirty genera of the Figure.

**Figure 5:**
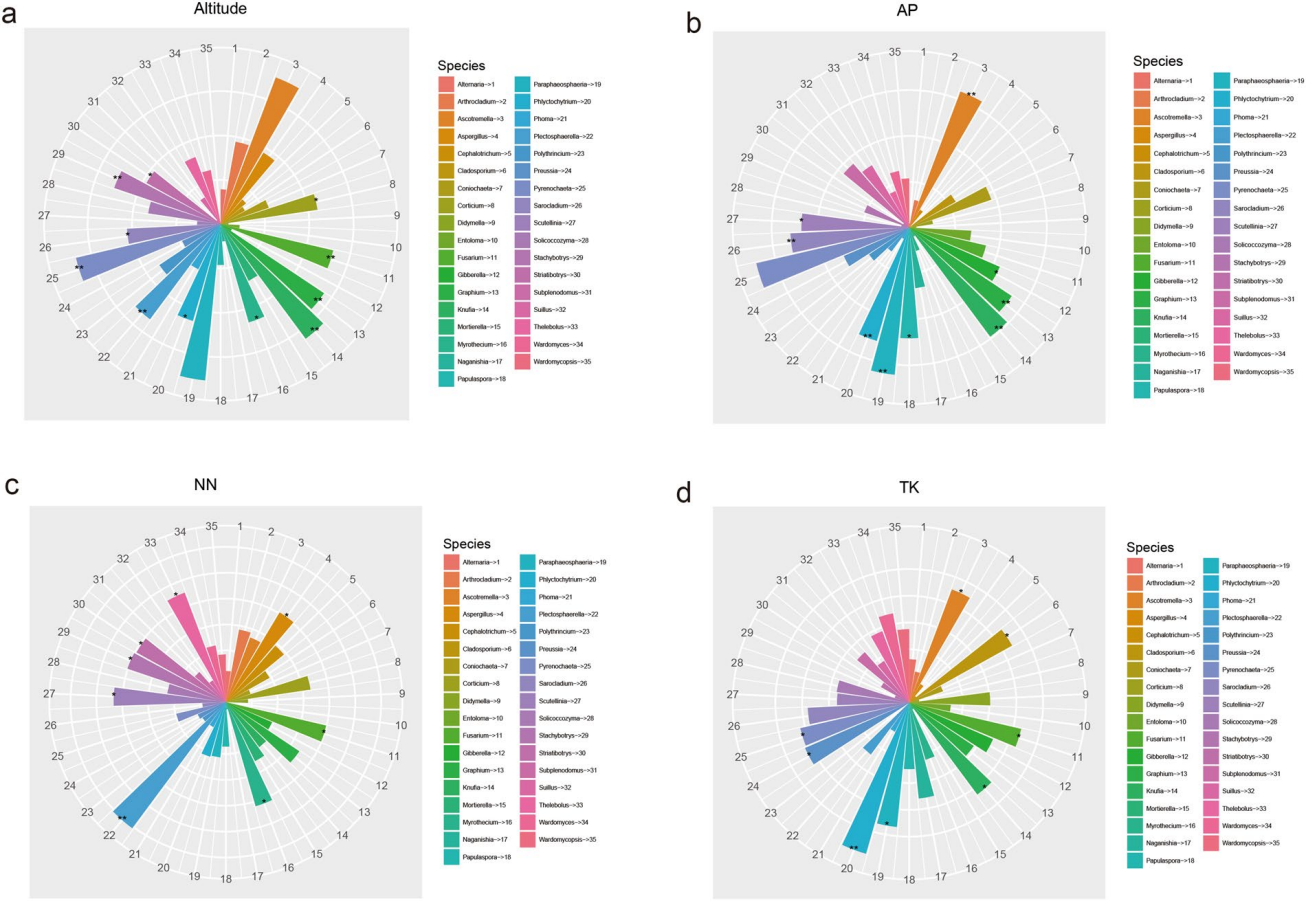
Spearman correlation analysis was used to evaluate the relationship between abiotic factors ((**a–d**) respectively represent Altitude, AP, NN, and TK) and fungal communities (genera). Description: p < 0.5 represents a significant correlation, (*) p < 0.01 is very significant (**), Positive correlation (+), Negative correlation (−). Abbreviations: phenyl group (PH),Total Organic Carbon (TOC), Total nitrogen (TN), Total phosphorus (TP), Total kalium (TK), Nitrate nitrogen (NN), Ammoniacal nitrogen (AN), Available phosphorus (AP), Total salt (TS).

OTUs and species classification analysis found that in the rhizosphere and non-rhizosphere soils of Ferula sinkiangesis, Polythrincium, Mortierella, Striatibotrys, Pyrenochaeta, Schizophyllum, Gibberella, Alternaria, Myrothecium, Arthrocladium and Graphium were relatively more enriched than other genera. Among the top thirty genera, Polythrincium, Mortierell and Striatibotrys were more highly enriched than the other genera, with Polythrincium having the highest abundance (Fig. 4). Gradually, Spearman correlation analysis showed a significant correlation between abiotic factors and fungal communities. We screened four abiotic factors associated with the majority of fungal communities, including altitude, AP, NN, and TK. Amongst them, altitude showed a significant positive correlation with Ascotremella, Fusarium, Graphium, Knufia, Paraphaeosphaeria, Phlyctochytrium, Pyrenochaeta, Sarocladium, Stachybotrys and Striatibotrys, and a significant negative correlation with Corticium, Myrothecium and Plectosphaerella (Fig. 5a). AP showed a significant positive correlation with Ascotremella, Gibberella, Graphium, Knufia, Paraphaeosphaeria, Phlyctochytrium, Pyrenochaeta, Sarocladium and Scutellinia, and a significant negative correlation with Scutellinia (Fig. 5b). NN showed a significant positive correlation with Scutellinia, Thelebolus, Stachybotrys, Myrothecium and Striatibotrys. Plectosphaerella, and a significant negative correlation with Aspergillus and Fusarium (Fig. 5c). TK showed a significant positive correlation with Phlyctochytrium, Ascotremella, Cladosporium, Knufia, Fusarium, Preussia, Paraphaeosphaeria and Pyrenochaeta (Fig. 5d). The relationship between all abiotic factors and fungal genus (Supplementary Fig. S3).

### Different biomarkers are present in the rhizosphere and non-rhizosphere at different soil depth and sites

**Figure 6:**
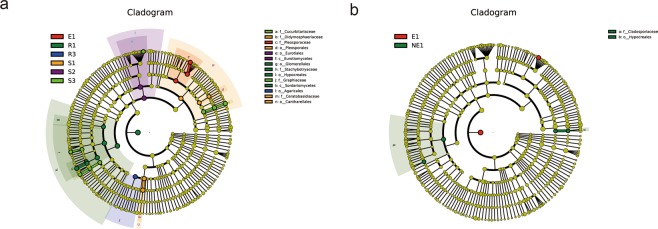
LDA Effect Size (LEfSe) analysis between groups. Biomarkers with statistical differences in different regions and depths (**a**) and Biomarker with statistical difference at 0–5 cm depths (**b**). Description: In the evolutionary branch diagram, the circle radiating from inside to outside represents the classification level from the gate to the genus (or species). Each small circle at a different classification level represents a classification at that level, and the diameter of the small circle is proportionate to the relative abundance. Coloring: species with no significant differences are uniformly colored yellow. Red nodes indicate the microbial group playing an important role and green nodes indicates an important role. Microbial Populations, if one group in the figure is missing this indicates that no significant differences are observed in the group. The name of the species represented by letters is shown in the legend (right side).

LDA effect size (LEfSe) analysis revealed different biomarkers in the rhizosphere at different soil depths from the same site. For example, in the rhizosphere of the Ferula sinkiangesis in the third site, enrichment of didymosphaeriaceae, Ceratobasidiaceae, Plecosporales and Cantharellales were significant in 0–5 cm soil, enrichment of eurotiomycetes and eurotiales were significant in deep soil (5 cm–15 cm) and enrichment of cucurbitariacea and graphiaceae were significant at a depth of 15–40 cm (Fig. 6a). Biomarkers of the rhizospheric area differed according to location at a depth of 0–5 cm. For example, at a soil depth of 0–5 cm, the accumulation of pleosporaceae in site 1 was significant. In site 2 Glomerellales, Stachybotryaceae, Hypocreales and Sordariomycetes were significant. In site 3, Didymosphaeriaceae, Pleosporales, Ceratobasidiaceae and Cantharellales were 144 abundant (Fig. 6a). At the same location, the enrichment of species in rhizosphere and non-rhizosphere soils at a depth of 0–5 cm also differed. For example, at a soil depth of 0–5 cm at site 1, Cladosporiaceae and Hypocrees enrichment in the non-rhizosphere soil was significant, and Pleosporacea enrichment in the rhizosphere soil was significant (Fig. 6b).

## Discussion

Microorganisms participate in soil food webs as decomposers^50,51^. In this study, we found that the dominant fungi in the root soil of *Ferula Sinkiangensis* were Ascomycota, Basidiomycota, Chytridiomycota, Mortierellomycota, Glomeromycota, Rozellomycota, Zoopagomycota, Olpidiomycota, Mucoromycota and Blastocladiomycota. Amongst them, Ascomycota, Basidiomycota, and Chytridiomycota had absolute quantitative advantages. These conclusions are consistent with the results of the dominant position of ascomycetes observed by Chen and colleagues in the grassland soil fungal community, and the dominant results of the ascomycetes identified by Hugoni and colleagues in the soil fungal community of Gramineae^28,52^. Ascomycota is considered a key player in the decomposition of soil organic matter^53,54^ and largely dominates the active fungal community through its involvement in root exudation assimilation and SOM degradation^28^. In addition, Ascomycetes, Blastocladiomycota, Mortierellomycota, Olpidiomycota and Chytridiomycota can be saprophytic on the residual limbs of plants and animals and decompose their remains^55–59^. Glomeromycota can form arbuscular mycorrhiza with plants and are able to absorb nutrients directly^60^. Rozellomycota and Zoopagomycotan gain nutrients from invading plants^61^. This indicates that fungi in the rhizosphere niche perform a variety of tasks. This is supported by studies from Ballhausen and coworkers. A saprophytic rhizosphere term defines the area affected by saprophytic mycelium. Its contribution to the food web is underestimated, particularly from the view of saprophytic fungi^19^. This consequently underestimates the status of fungi in the ecological cycle.

Linear Discriminant Analysis effect size (LEfSe) analysis showed significant differences between rhizosphere and non-rhizosphere biomarkers in different regions at a depth of 0–5 cm (Fig. 6b). We believe that these differences are due to natural environments in the regions, the trend effect of different environmental factors on the microorganisms^62,63^ and explains why microorganisms in the topsoil are more susceptible to the environment^64–66^. We observed different markers at varying depths (Fig. 6a), which were roughly divided into three layers. (1) the surface layer of plant litter accumulation; (2) the bottom layer of root exfoliation accumulation; and (3) the middle layer. Due to nutritional trends, the fungi that feed on cellulose and lignin accumulate on the surface and bottom layer, as the surface layer is sensitive to various environmental factors to which the fungi can adapt. The secretion of sugars and simple compounds permits bacteria to be enriched to high levels, at which point more competitive fungi become enriched. In the middle layer, it is difficult to decompose lignin, so lignin-feeding fungi become enriched, resulting in different biomarkers at each soil depth. However, the root system environment is more complex and requires experimental evidence. In addition, this study indicates that no significant differences between the rhizosphere and non-rhizosphere below 0–5 cm exist. We suspect that this is due to the influence of volatile components in the roots of *Ferula sinkiangesis*, The volatile substances of *Ferula sinkiangesis* are related, permitting a new layer of experimental design.

Dominant soil microbes (genus) have specific relationships to abiotic factors. In addition to altitude, TK, AP and NN have significant interrelationships with the majority of dominant microbes and other non-biological factors identified in this study. Some correlation with microbes also exists. For example, pH negatively correlates with mortierella and Arthrocladium, but positively correlates with Gibberella and Alternaria. There is also a significant positive correlation between TPand Alternaria, Paraphaeosphaeria, Knufia and Coniochaeta. A significant positive correlation between AN and Solicoccozyma and Papulaspora also occurred, whilst a significant negative correlation with Ascotremella, Knufia, Paraphaeosphaeria and Pyrenochaeta was observed. There was a significant positive correlation between TN and Pyrenochaeta and Knufia and between TS and Wardomycopsis No correlation between TOC and the dominant microbial genus occurred (Supplementary Fig. 3). Understanding this inter-relationship between abiotic factors and microorganisms is of great significance to reveal themechanistic interactions between plants and microorganisms, and to understand the role of microorganisms in this niche.

A significant correlation exists between abiotic factors and soil microbial community diversity and abundance. Yao qin and colleagues demonstrated that soil pH and TK have significant effects on the microbial community structure, and artificially increasing biochars can influence the properties of the soil^67^. In this study, we found significant positive correlation between TK and fungal biodiversity, and so identified that K fertilizers in the habitat soil of *Ferula Sinkiangensis*can affect the fungal community, indirectly optimizing the soil structure. The purpose of this is to improve the growth environment of the ferula. The investment in TK is not unlimited, and a threshold is likely to be reached and various factors are likely to interact. The correct allocation of the investment requires further experiments to explore the most reasonable input.

In summary, this study provides information on the rhizosphere fungal community and the diversity of *Ferula Sinkiangensis*. Moreover, different biomarkers were identified at varying depths, which may be related to the medicinal components of *Ferula Sinkiangensis*. We also identified a link between environmental factors and fungal community diversity, in addition to fungal genus and gates. We hope that by mastering these relationships, we can artificially intervene and regulate the diversity of fungal communities to improve soil structure and ferlity. This information is useful for the protection and commercial cultivation of *Ferula Sinkiangensis*.

## Supplementary information

Supplementary material.
