# Supplementary material for: High-throughput sequencing reveals the diversity and community structure of rhizosphere fungi of Ferula Sinkiangensis at different soil depths
Source: Sci Rep. 2019 Apr 25;9:6558. doi: 10.1038/s41598-019-43110-z (PMC6484027; doi:10.1038/s41598-019-43110-z)
Supplement: Supplementary file 1 — Supplementary material. [file 41598_2019_43110_MOESM1_ESM.pdf]

High-throughput sequencing reveals the diversity and community structure of rhizosphere fungi of *Ferula Sinkiangensis* at different soil depths

Tao Zhang[1], Zhongke Wang[1], Xinhua LV[1],Yang Li[1],Li Zhuang[1]*

[1] College of Life Sciences, Key Laboratory of Xinjiang Phytomedicine Resource Utilization, Ministry of Education, Shihezi University, Xinjiang Shihezi, 832003, China.

*** Correspondence: Li Zhuang** email:3033573705@qq.com

Keywords: Soil fungi; Rhizospheric and non-rhizospheric soil; High-throughput sequencing; Advantage fungi group; Abiotic factor.

supplementary materials

Supplementary Figure 1

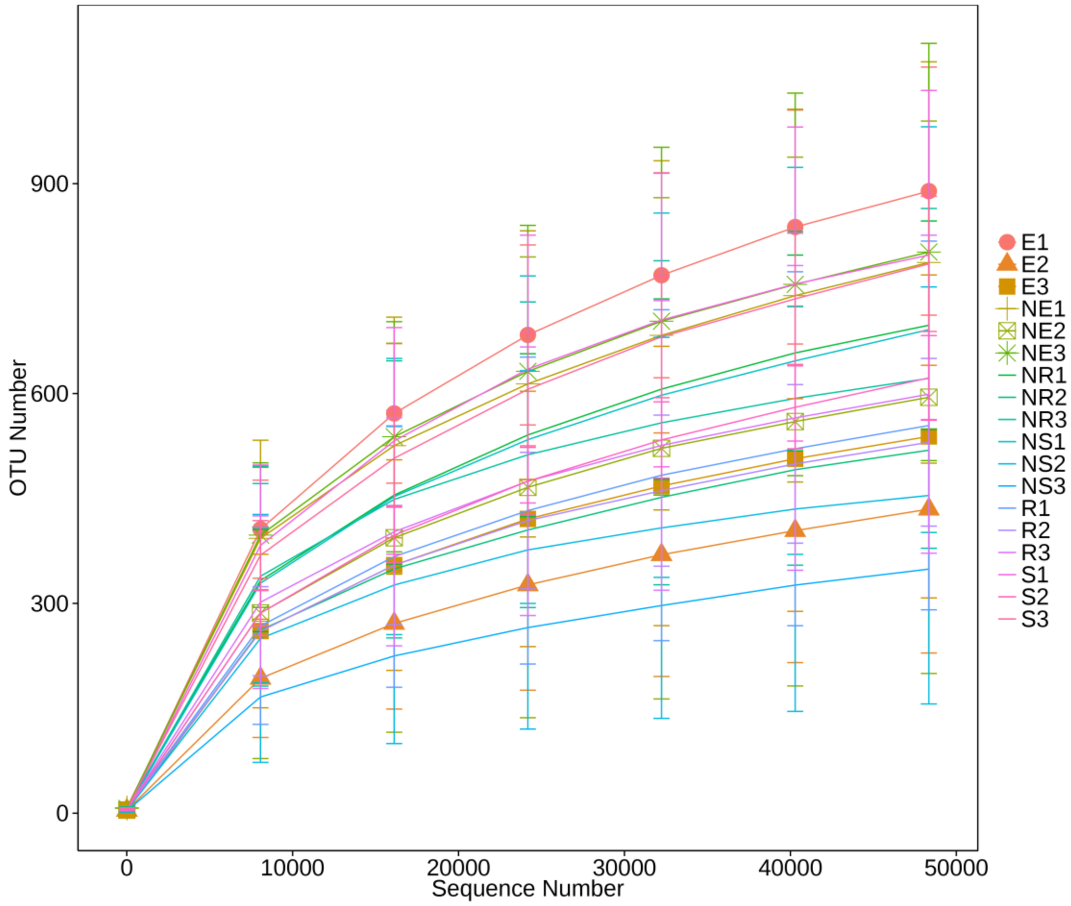

Species rarefaction curves. Description: The abscissa is the number of sequencing sequences randomly selected from a sample, and the ordinate is the number of OTU

that can be constructed based on the number of sequencing sequences, which is used to reflect the sequencing depth. Different samples are represented by different color curves.

Supplementary Figure 2

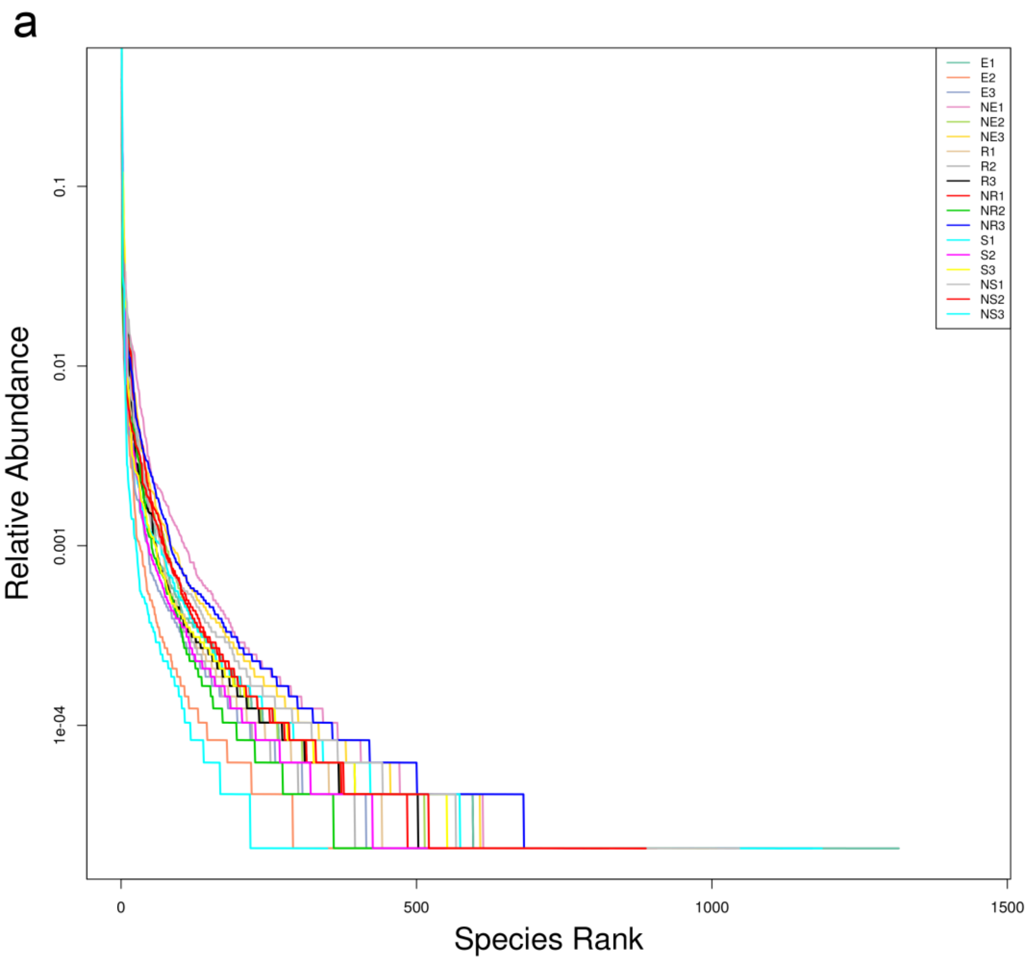

Species rank abundance curve. Description: The abscissa is the ordinal sorted by OTUs abundance, and the ordinate is the relative abundance of OTUs. Different samples are represented by broken lines of different colors.

Supplementary Figure 3

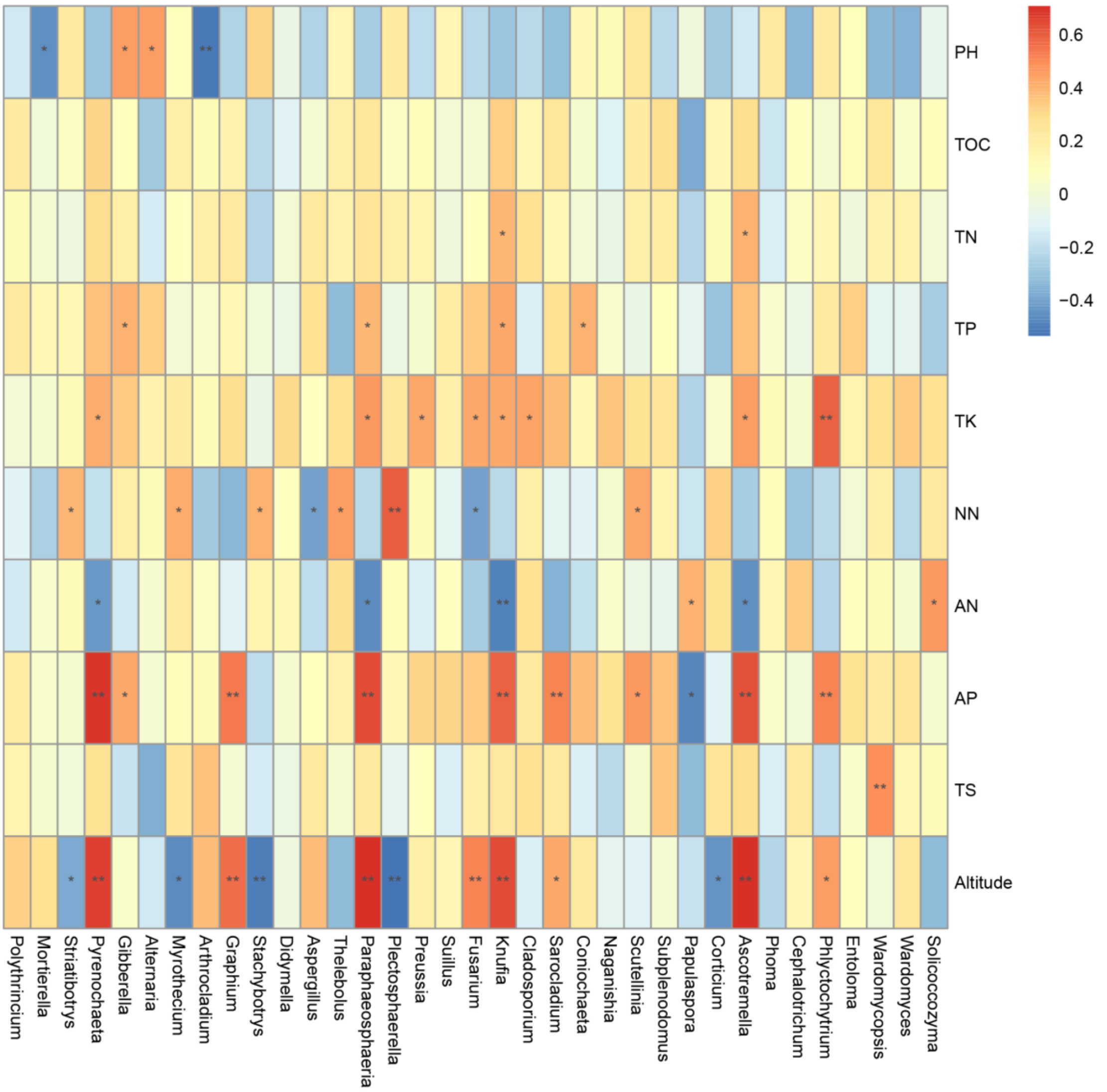

Spearman correlation analysis to assess the relationship between abiotic factors and fungal communities. Description: $p<0.5$ represents a significant correlation, (*) $p<0.01$ is very significant (**), Positive correlation (+), Negative correlation (-). Abbreviations: phenyl group (PH),Total Organic Carbon (TOC), Total nitrogen (TN), Total phosphorus (TP), Total kalium (TK), Nitrate nitrogen (NN), Ammoniacal nitrogen (AN), Available phosphorus (AP), Total salt (TS),Abundance Coverage based Estimator (ACE), Shannon's diversity index (Shannon). Description: $p<0.5$ represents a significant correlation (*) $p<0.01$ is very significant (**).

Supplementary Table 1

Table 1. Description of three sampling locations in Yining, Xinjiang, China.

site	Altitude	Longitude	Latitude
Byshdun	993m	E82.083359	N43.723121
Ferulic Beach	1136m	E82.109673	N43.740984
Ferulic protected areas	1093m	E82.103653	N43.754301
